# Advances in Immune Checkpoint Inhibitors for Advanced Hepatocellular Carcinoma

**DOI:** 10.3389/fimmu.2022.896752

**Published:** 2022-06-10

**Authors:** Yue Chen, Haoyue Hu, Xianglei Yuan, Xue Fan, Chengda Zhang

**Affiliations:** ^1^Department of Pathology, Beijing Shijitan Hospital, Capital Medical University, Beijing, China; ^2^Department of Medical Oncology, Sichuan Cancer Hospital and Institute, Sichuan Cancer Center, Medicine School of University of Electronic Science and Technology, Chengdu, China; ^3^Department of Gastroenterology, West China Hospital of Sichuan University, Chengdu, China; ^4^Department of Gastroenterology, The Third Hospital of Mianyang (Sichuan Mental Health Center), Mianyang, China

**Keywords:** hepatocellular carcinoma, immune checkpoint inhibitors, tyrosine kinase inhibitors, vascular endothelial growth factor, tumor microenvironment

## Abstract

Hepatocellular carcinoma (HCC) is usually diagnosed in an advanced stage and has become the second deadliest type of cancer worldwide. The systemic treatment of advanced HCC has been a challenge, and for decades was limited to treatment with tyrosine kinase inhibitors (TKIs) until the application of immune checkpoint inhibitors (ICIs) became available. Due to drug resistance and unsatisfactory therapeutic effects of monotherapy with TKIs or ICIs, multi-ICIs, or the combination of ICIs with antiangiogenic drugs has become a novel strategy to treat advanced HCC. Antiangiogenic drugs mostly include TKIs (sorafenib, lenvatinib, regorafenib, cabozantinib and so on) and anti-vascular endothelial growth factor (VEGF), such as bevacizumab. Common ICIs include anti-programmed cell death-1 (PD-1)/programmed cell death ligand 1 (PD-L1), including nivolumab, pembrolizumab, durvalumab, and atezolizumab, and anti-cytotoxic T-lymphocyte-associated protein 4 (CTLA4), including tremelimumab and ipilimumab. Combination therapies involving antiangiogenic drugs and ICIs or two ICIs may have a synergistic action and have shown greater efficacy in advanced HCC. In this review, we present an overview of the current knowledge and recent clinical developments in ICI-based combination therapies for advanced HCC and we provide an outlook on future prospects.

## Introduction

Liver cancer is a global health burden with an increasing incidence and a leading cause of cancer-related deaths (1). Hepatocellular carcinoma (HCC) is the most common type of liver cancer, and 72% of cancer-related death cases are observed in Asia (2). Most cases (80-90%) of HCC can be considered prototypical inflammation-driven cancers for the backdrop of chronic liver injury/cirrhosis caused by hepatitis B virus (HBV) or hepatitis C virus (HCV) infections, alcohol abuse, obesity, and aflatoxin B1 (3). The high mortality of HCC is attributed to an advanced-stage presentation and a high prevalence of liver dysfunction. For delayed diagnosis, postsurgical recurrence and metastasis, there is a poor 5-year survival rate of less than 50% (4).

The Barcelona Clinic Liver Cancer (BCLC) system is the most commonly recommended staging system for HCC. Based on the underlying liver function evaluated by the Child–Pugh score and the performance status, HCC patients can be divided into five classes, including BCLC stage 0, A, B, C and D (5). This classification is associated with the treatment strategies and prognosis of HCC. For early-stage (BCLC stage 0 and A) HCC patients, those with solitary nodules less than 3 cm or multiple nodules less than 3 cm limited in the liver with preserved liver function and without macrovascular invasion, curative approaches, such as surgical resection, ablation, and liver transplantation could be effective. For large, multinodular without vascular invasion intermediate-stage HCC (BCLC stage B), transcatheter arterial chemoembolization (TACE) is the preferred treatment option if liver function is preserved. Unfortunately, most patients are diagnosed at a relatively advanced stage (BCLC stage C) with a poor prognosis and a survival time of less than 1 year. In this state, tumors have expanded outside the liver or vascular invasion or liver dysfunction (6). Due to the strong and extensive resistance of chemotherapy, as well as the increasing toxicity for the underlying altered liver function, the use of cytotoxic agents is frequently restricted in HCC. Clinical trials using doxorubicin in combination with cytotoxic chemotherapy have proven that there are low response rates with no survival benefit (7). Therefore, systemic therapies are required at an advanced stage. For patients in the terminal stage (BCLC stage D) with poor liver function, supportive care is required when they are not considered suitable for transplantation (8).

The common pathophysiological features of hypervascularity and vascular abnormalities include sinusoidal capillarization and overexpression of proangiogenic growth factors, such as vascular endothelial growth factor (VEGF) and platelet-derived growth factor (PDGF) in HCC. In recent decades, anti-angiogenesis has attracted attention as a potential therapeutic target (9). Sorafenib, an oral small molecule multityrosine kinase inhibitor (TKI) that can suppress angiogenesis, exerts an anticancer effect by inhibiting vascular endothelial growth factor receptor (VEGFR) and fibroblast growth factor receptor (FGFR) (10). In 2007, two phase III trials (one is the SHARP trial in Europe and the USA, one is the ORIENTAL trial in Asia-Pacific regions) showed promising results that sorafenib significantly prolonged the survival of advanced-stage HCC patients compared with the placebo (11, 12). Based on the results of these two clinical trials, sorafenib was recommended as a first-line targeted agent for advanced HCC worldwide in the 2008 NCCN guidelines (10). Even for transplant recipient patients with unresectable HCC, sorafenib is generally well-tolerated and associated with improved overall survival (OS) (13, 14). Lenvatinib, another oral small molecule multi-TKI that inhibits tumor angiogenesis and growth, was found to be no less effective than sorafenib. Hence, lenvatinib therapy became the second recommended first-line targeted molecular therapy in the 2019 NCCN guidelines (15). Other multitarget TKIs, regorafenib and cabozantinib, were recommended as second-line agents for HCC patients who progressed on sorafenib treatment in the 2017 and 2019 NCCN guidelines, respectively (15, 16). Ramucirumab, a recombinant IgG1 monoclonal antibody (mAb) and an inhibitor of VEGFR2, showed efficacy after sorafenib among advanced patients with elevated levels of α-fetoprotein (AFP) (17). In view of this, ramucirumab was included in the second-line therapy in the 2019 NCCN guidelines (15). However, low objective response rates (ORRs), an improvement in OS of only 2-3 months, resistance, and cancer progression after standard treatment, regardless of first- and second-line settings, were observed, and therefore, more efficacious therapeutics should be explored (18).

HCC is a chronic inflammation-induced type of cancer that expresses various antigens that can mediate immune responses. Over the past decade, immune-based therapies that modulate the balance of immune homeostasis have been increasingly explored and have shown beneficial outcomes in HCC (19). Immune checkpoints include coinhibitory receptors on T cells and their ligands on tumor cells and stromal cells in the tumor microenvironment (TME). Immune checkpoint inhibitors (ICIs) prevent the inactivation of T cells by blocking interactions between checkpoint proteins and their ligands, such as those mediated by programmed cell death-1 (PD-1)/programmed cell death ligand 1 (PD-L1), cytotoxic T-lymphocyte-associated protein 4 (CTLA4), T-cell immunoglobulin, mucin domain containing-3 (TIM3), and lymphocyte-activation gene 3 (LAG3), thereby exerting antitumor effects (20, 21). However, not all patients (especially in the era of pre-liver transplantation) with HCC respond to immunotherapy, and more importantly, the ORR is low, and OS does not significantly improve with single-agent immunotherapy (22, 23). Given these data, more effective combination therapies for the treatment of HCC are explored, including ICIs combined with other ICIs, TKIs, anti-VEGFs, and other agents (24). In recent years, the emergence of combination therapies using multi-ICIs or ICIs with antiangiogenics represents the main avenue for the treatment of advanced HCC (5, 25). The objective of this review is to focus on the current knowledge of ICI monotherapy or in combination with other ICIs or molecularly targeted therapies (TKIs or anti-VEGFs) in advanced HCC and to provide an outlook on future prospects.

## Immune Microenvironment of the Liver

**Figure 1:**
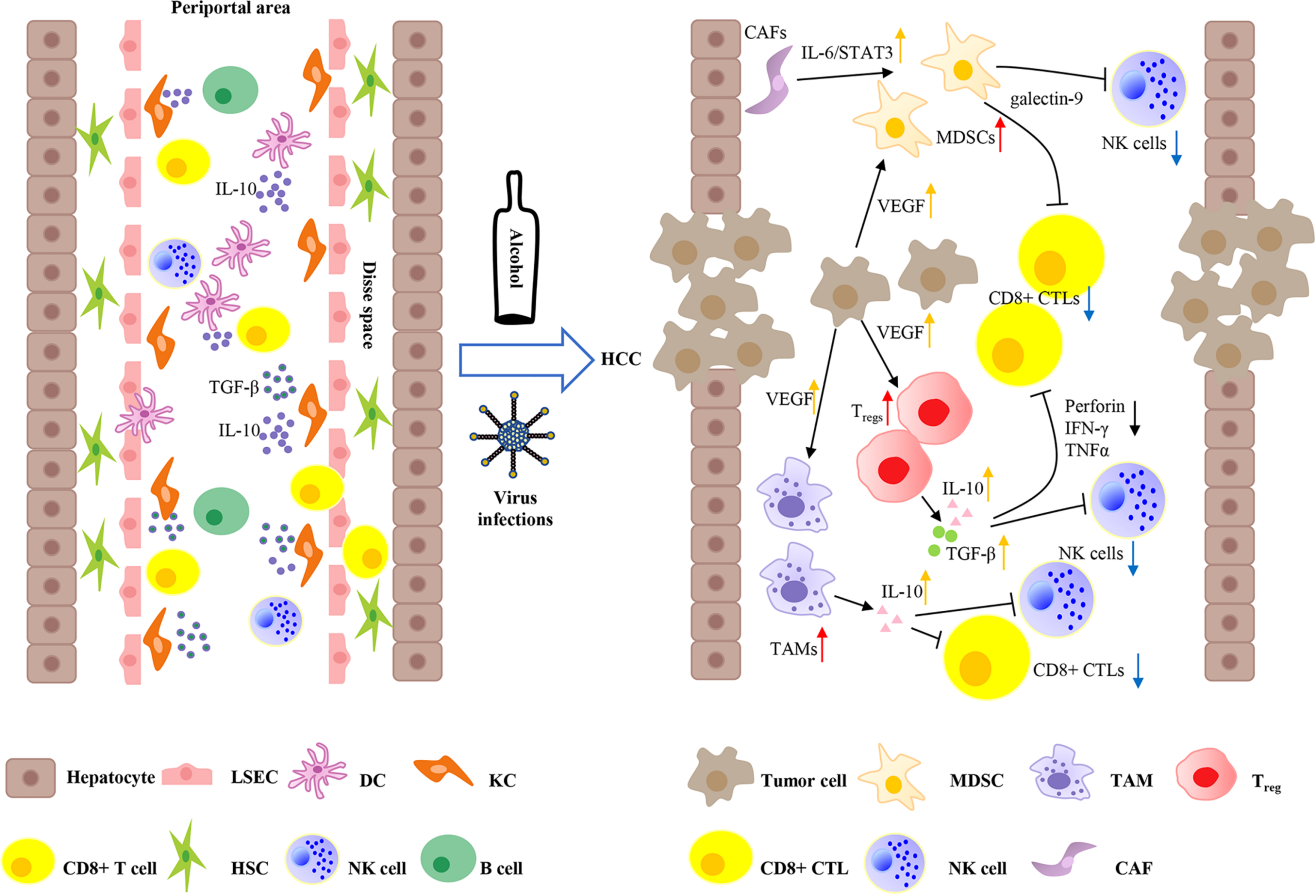
Immune microenvironment of liver and tumor microenvironment of hepatocellular carcinoma. The liver not only regulates immune responses, but also maintains immune tolerance to self and foreign antigens. Liver sinusoidal endothelial cells (LSECs) line the liver sinusoid wall that controls the exchange of materials between hepatocytes and blood. Kupffer cells (KCs) and liver resident dendritic cells (DCs) can access to the Disse space to get in touch with hepatocytes and hepatic stellate cells (HSCs). KCs are key regulators of tolerance by expressing a large amount of IL-10 and transforming growth factor beta (TGF-β). Moreover, liver DCs produce elevated amounts of IL-10, resulting in immune tolerance. Continuous hepatitis B virus (HBV) or hepatitis C virus (HCV) infections, and alcohol abuse can lead eventually to the development of HCC. HCC is hypervascularity and overexpresses VEGF, which can recruit several inhibitory cells, such as myeloid-derived suppressor cells (MDSCs), tumor-associated macrophages (TAMs), and regulatory T cells (T_regs_) to form an immunosuppressive microenvironment. In addition, HCC-related cancer-associated fibroblasts (CAFs) can induce the differentiation of MDSCs by IL-6/STAT3 signaling. T_regs_ can produce suppressive cytokines IL-10 and TGF-β to impair the inflammatory functions of CD8+ cytotoxic T lymphocytes (CTLs) and natural killer (NK) cells through inhibiting tumor necrosis factor-α (TNF-α), interferon-γ (IFN-γ), and the release of perforin. Furthermore, T_regs_ and TAMs also secrete IL-10 to attenuate the capacity of CD8+ CTLs and NK cells. Moreover, MDSCs express TIM3 ligand galectin-9 and induce T-cell apoptosis.

The liver is an organ with metabolic function and immune regulatory function. Liver cells are commonly exposed to food antigens and gut pathogens in terms of the dual supply of arterial and portal systemic blood (26). Therefore, the liver not only regulates immune responses but also has the ability to maintain immune tolerance to self and foreign antigens. This tolerogenic environment is maintained by specialized immunocytes, including Kupffer cells (KCs), liver resident dendritic cells (DCs), liver sinusoidal endothelial cells (LSECs), hepatic stellate cells (HSCs), natural killer (NK) cells, and innate T and B cells (27). Among them, KCs, DCs, HSCs, and LSECs are antigen-presenting cells (APCs). DCs (conventional APCs) exist in multiple subtypes with different functions. Under physiological conditions, in the hepatic microenvironment, DCs appear as a tolerogenic phenotype and can secrete an array of immunosuppressive cytokines, including interleukin 10 (IL-10), prostaglandin E2 (PGE2), and indoleamine 2,3-dioxygenase (IDO), which can promote regulatory T cell (T_reg_, derived from naive CD4+ T cells) activation, thus playing an inhibitory role in innate immune responses (28). Under homeostatic conditions, non-conventional APCs (KCs, LSECs, and HSCs) in the liver are known to act as weak T-cell activators due to low expression of major histocompatibility complex (MHC) molecules and APC activation markers CD80 and CD86 (29). KCs eliminate high-affinity antigen-specific CD8+ T cells in the liver and express heightened amounts of IL-10 and transforming growth factor beta (TGF-β) to promote the activation of T_regs_ (**Figure 1**) (30, 31). In addition, a variety of immune checkpoint proteins limit T-cell hyperactivation in physiological circumstances. T cells express CTLA4, PD-1, LAG3, and TIM3, which interact with ligands on APCs (such as PD-L1) and play a key role in immune tolerance in the liver (32).

## Tumor Microenvironment of HCC

It is an immense challenge to produce immune tolerance or immune response by distinguishing between benign foreign antigens and pathogenic antigens. Failure to respond to HBV and HCV infections would markedly induce immunosuppression and impair immune surveillance, which increases the risk of chronic infections and ultimately gradually develops into HCC (33). The TME of HCC is composed of immune cells (cytotoxic CD4+ T cells, CD8+ T cells, and NK cells), abundant immunosuppressive cells, such as T_regs_, myeloid-derived suppressor cells (MDSCs), tumor-associated macrophages (TAMs), stromal cells, the extracellular matrix (ECM), blood vessels, tumor cells, and lymphatic vessels, which play an important role in tumor survival, proliferation, invasion, and metastasis (34). Immune cells recognize and kill cancer cells. Moreover, deficiencies and malfunctioning of immune cells can influence the balance of the TME and lead to an immunosuppressive microenvironment. Several factors, including immunity suppression, chronic inflammation, and the decreased recognition of cancer cells have been suggested to play a role in promoting tumor antigen tolerance, which induces hepatocarcinogenesis (35). In a number of recent clinical trials, it was highlighted that the onset of HCC may be favored by alterations in cytokine levels as well as in immune cell function and number. IL-6 is a pleiotropic cytokine that exerts its biological effects mainly through the IL-6/STAT3 signaling pathway. IL-6 is abundantly present in the TME, and an abnormally activated IL-6/STAT3 signaling pathway can play a role in the occurrence and development of HCC by affecting tumor cell proliferation, migration, invasion, angiogenesis, and apoptosis (36, 37). IL-10 and TGF-β are important regulatory cytokines of hepatocytes. Moreover, in addition to overcoming the tumor suppressor effect of hepatocytes, the mechanism of action of tumor cell development involves other pathways related to IL-10 and TGF-β, such as epithelial-mesenchymal transition (EMT) and suppressing IFN-γ production, which contributes to tumor progression and metastasis (38). The TME is shaped by complex interactions between tumor cells and immune cells. HCC has a high degree of malignancy and the poor survival rate of patients is closely related to an imbalance of the immune microenvironment, the breakdown of immune system surveillance, and the suppression of host immune system responses. These components synergistically construct an immunosuppressive microenvironment in HCC *via* a variety of mechanisms (**Figure 1**) (19).

### Immunosuppressive Cells in the TME of HCC

MHC I/II is usually functionally depleted in HCC, is unable to activate T cells, and downregulates the expression of the costimulatory molecular receptor B7 family (such as B7.1/B7.2), leading to immune escape, which is a prerequisite for tumorigenesis (39). Low expression of MHC-I (binding to cytotoxic CD8+ T cells) and high expression of MHC-II (binding to immunosuppressive CD4+ T cells) is the reason for immune escape in terms of the failure of antigen presentation related to HCC. The result is that a large number of immunosuppressive cells are recruited into the TME of HCC (40).

#### Regulatory T Cells (T_regs_)

T_regs_ play a pivotal role in antitumor suppression and are mainly derived from peripheral blood or resident naive CD4+ T lymphocytes, and are recruited by the CC chemokine receptor 6 (CCR6)-CC chemokine ligand 20 (CCL20) axis (41). The differentiation of T_regs_ from CD4+ T cells requires the action of cytokines IL-2 and TGF-β, followed by the production of the suppressive cytokines IL-10 and TGF-β by expressing the transcription factor Foxp3, which in turn promotes further differentiation and suppresses inflammatory functions (42, 43). Compared with normal liver tissue, the proportion and number of CD4+CD25+ T_regs_ are markedly increased in HCC. Among these, CD4+ CD25+ Foxp3+ subtype T_regs_ have been found to suppress CD8+ cytotoxic T lymphocyte (CTL) activation and disable the killing capacity of CTLs by inhibiting tumor necrosis factor-α (TNF-α) and interferon-γ (IFN-γ) and the release of granzyme A, B (GrA, B), and perforin (44, 45). Another mechanism is the disruption of antigen presentation by downregulation of CD80 and CD86 expression in DCs and direct lysis of APCs *via* GrA and GrB (46, 47).

#### Myeloid-Derived Suppressor Cells (MDSCs)

MDSCs are immature myeloid cells that originate from the bone marrow that are increased in HCC and upregulate the expression of immune suppressive factors to suppress antitumor immunity in HCC (48). HCC-related cancer-associated fibroblasts (CAFs), which are components of the extracellular matrix in the TME, can induce MDSC differentiation from peripheral blood monocytes *via* IL-6/STAT3 signaling (49). In a previously established mouse model, it was demonstrated that granulocyte-macrophage colony-stimulating factor (GM-CSF), IL-6, VEGF, and other tumor-associated cytokines could promote the accumulation and migration of MDSCs. Recent evidence has shown that a cell cycle-related kinase (CCRK) unique to HCC can also induce MDSC infiltration into the TME by promoting the expression of IL-6 by activating the zeste homolog 2 (EZH2)/nuclear factor-κB (NF-κB) signaling pathway (50). In addition, local hypoxia (a crucial factor in the TME of solid tumors) is another key factor for the recruitment of MDSCs with the action of the chemokine (C-C motif) ligand 26 (CCL26)/CX3CR1 pathway (51). MDSCs exert continuous immune-suppressive effects by inducing CD4+ CD25+ Foxp3+ T_regs_, damaging CD8+ T cells, expanding immune checkpoint signaling and inhibiting NK-cell cytotoxicity (52, 53). MDSCs express the TIM3 ligand galectin-9 and induce T-cell apoptosis (54). Furthermore, PD-L1 expression can be induced by MDSCs in concert with KCs in advanced HCC, which mediates the inhibition of NK-cell cytotoxicity (55).

#### Tumor-Associated Macrophages (TAMs)

TAMs are predominant tumor-infiltrating leucocytes and vary depending on the cancer type (56). In HCC, TAMs arrive from CCR2+ inflammatory monocytes after the induction of the HCC-derived cytokines IL-4, CCL2, CXCL12, and others. Based on the state of macrophage activation, TAMs can be divided into two polarizing phenotypes, M1 and M2. M1 is the classical phenotype and activated by interferon-α, β or γ (IFNα/β/γ), which induces antitumor immune responses. In contrast, M2 is the alternative phenotype and activated by IL-4 and IL-10, which stimulate tumor promotion and metastasis by various mechanisms (57, 58). This suggests the presence of both antitumorigenic (M1) and protumorigenic (M2) macrophages in HCC, and the balance of M1/M2 is regulated by various TME components. TAMs contribute to malignant progression and metastasis by the production of IL-6, epithelial-to-mesenchymal transition (EMT) and immunosuppression (59). TAMs are highly associated with immune checkpoint molecules, such as PD-1/PD-L1, CTLA4, and TIM3, to exert immune inhibitory regulation. TAMs in the tumor stroma of HCC secrete pivotal cytokines (e.g., NF-α, IL-6, IL-23) and expand IL-17-producing CD4+ T helper 17 cells (Th17), which inhibit antitumor immunity by upregulating PD-1 and CTLA-4 (60). Moreover, TAMs can directly promote T_reg_ expansion *via* surface expression of PD-L1. In addition, TAMs in HCC promote the expression of TIM3 by TGF-β stimulation, thereby ultimately facilitating tumor progression and immune tolerance (61).

#### CD8+ Cytotoxic T Lymphocytes (CTLs)

Naive CD8+ T cells (without cytotoxic activity) can become CTLs when they receive a signal from costimulatory molecules and then have the ability to protect against APCs. CD8+ CTLs can recognize abnormal cells, such as tumor cells by cooperating with helper T1 cells (Th1) and mediate antitumor immune responses by releasing perforin, granzyme, and TNF-α to damage tumor cells (48). However, the efficacy of CD8+ CTLs in HCC is functionally limited through a variety of mechanisms. Hypoxia, in conditions of an acidic environment (overload of lactic acid and low pH), lack the help of CD4+ T cells, and overabundant immunoregulatory molecules (IL-10, VEGF, IDO), may be responsible for restricted CD8+ CTL-specific cytotoxic responses (62). Unlike other TME immunosuppressive cells, the infiltration of CD8+ CTLs can be reduced by liver fibrosis (a striking feature of HCC) by disrupting CD8+ T-cell recognition of platelet-derived CD44 (63). Most CTLs are exhausted after their effect, but some remain memory killer cells that respond to the same tumor cells quickly when they are encountered in the future. In HCC, TOX, a novel T-cell exhaustion transcription regulator, is heavily overexpressed in CD8+ T cells, thereby suppressing cytotoxic effector and memory function (64). Notably, immune checkpoint signaling has recently been found to remarkably induce CTL exhaustion. PD-1/PD-L1 signaling is a crucial driver of CTL exhaustion (inhibition of T-cell survival and growth) in HCC and plays a role by blocking T-cell receptor (TCR) sequences through the PI3K/AKT pathway. CTLA-4 is upregulated after the activation of T cells and acts as a competitive antagonist of CD80 and CD86 in APCs and inhibits downstream AKT signaling, thereby ultimately exerting inhibitory effects (65). Other drivers of T-cell exhaustion include TIM3 and LAG3, which are expressed on CD8+ T cells and T_regs_ in HCC and lead to hypofunctional CD8+ responses by reducing CTL capacity (66, 67).

#### Natural Killer (NK) Cells

NK cells are innate immune cells with a high frequency (~30%) in the liver and a low frequency in peripheral blood. Upon NK-cell activation triggered by virus-infected cells and tumor cells, NK-cells function rapidly without antigen presentation (68). NK cells are crucial in maintaining the balance of immune defense/tolerance. The antitumor effect of NK cells is induced by secreting several killer cytokines (e.g., IFN-γ and TNF-α) and chemokines and by inducing tumor cell apoptosis *via* the Fas/FasL pathway as well as the release of cytotoxic granules (mainly perforin and granzyme) (69). In HCC, increasing evidence has shown that hypoxia can dysfunction the antitumor immunity of NK cells by utilizing TME immunosuppressive components to influence the switch of activating/inhibiting NK receptors (NKRs). For example, AFP (known to be overexpressed in HCC), especially when extended, decreased the expression of natural killer group 2, member D (NKG2D), an activating NKR, and negatively regulated NK-cell viability (70, 71). Other modulators in the TME, such as T_regs,_ release the cytokines IL-8, IL-10, and TGF-β to downregulate NKG2D ligand membrane expression in HSCs, which suggests tumor progression in HCC patients (72).

### Extracellular Matrix (ECM) in TME of HCC

Chronic liver inflammation/injury causes liver fibrosis, which is characterized by the continuous accumulation of ECM-producing myofibroblasts and results in the gradual substitution of liver parenchyma by fibrous or scar tissue and liver cirrhosis (73). In the physiological liver, quiescent HSCs localize in the space of Disse but are activated in myofibroblasts and secrete ECM components in the pathological liver (74). CAFs are most important components that form the ECM and promote EMT (normal epithelial cells transform into mesenchymal cells) in the TME. CAFs mostly stem from HSCs or bone marrow (BM)-derived activated mesenchymal stem cells (MSCs). CAFs can alter stiffness of the ECM, secrete cytokines, including epidermal growth factor (EGF), TGF-β, and PDGF, and in turn promote tumorigenesis of the liver. Moreover, CAFs have been found to indirectly promote HCC through crosstalk with immunosuppressive cells (mostly MDSCs and T_regs_) in the TME, and reduce immune surveillance (75). Specifically, MDSC production can be induced by CAFs through the IL-6/STAT3 signaling axis and secretion of stromal cell-derived factor (SDF)-1α. More recently, in several studies, it was demonstrated that MDSC differentiation from blood monocytes can be promoted by PGE2 secretion in a CD44-dependent manner (76). CAFs also caused T-cell hyporesponsiveness and an increased number of T_regs_, followed by inhibition of T-cell-mediated cytotoxicity (77). In summary, CAFs play a critical role in contributing to the occurrence of liver fibrosis and the progression of HCC in the TME.

### Cytokines in the TME of HCC

The abundance of cytokines in the TME of HCC can mediate intercellular crosstalk and have multiple other functions. Based on their function, these cytokines can be classified into two groups. One type involves immune response cytokines, including TNFα, IFN-γ, IL-1, and IL-17, and the other type involves immunosuppressive cytokines, including IL-10, IL-4, IL-8, and TGF-β (78, 79). IL-10 is produced by DCs, TAMs, T cells, and T_regs_ and is elevated in HCC, thereby directly impairing the function of NK cells and downstream CD8+ T cells. Moreover, IL-10 inhibits the stimulatory function of APCs and promotes elevation of PD-L1 in monocytes, thus exerting immune escape-promoting effects (56, 80). High expression of a large amount of TGF-β in the HCC TME is made possible by tumor cells, macrophages, and T_regs_. It not only attenuates the activation of DCs but can also trigger the activation of T_regs_ and impair the effector functions of T cells and NK cells to inhibit antitumor efficacy (81). Additionally, TGF-β increases TIM3 expression on TAMs, subsequently facilitating immune tolerance through the TNF-α/NF-κB signaling pathway (61). IFN-γ and TNFα are two pivotal cytokines that play a role in antitumor immune responses, while lower serum levels of these two cytokines were found in HCC. As mentioned above, the production of immunosuppressive cytokines, such as IL-10, TGF-β, and PD-L1 can suppress IFN-γ/TNFα production derived from NK cells or effector T cells (82).

## ICIs in HCC

**Figure 2:**
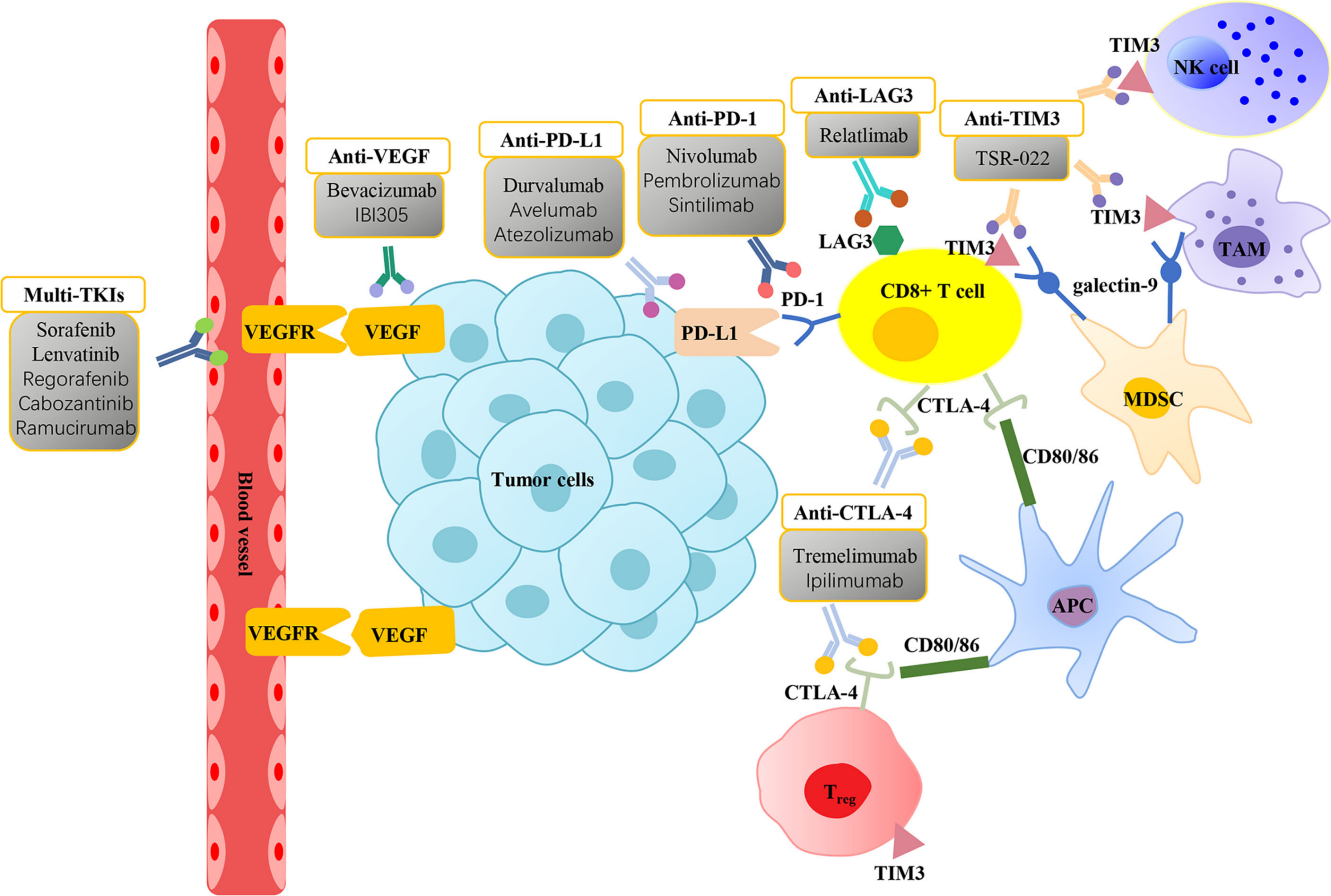
Molecularly targeted therapies and immune checkpoint inhibitors for the treatment of hepatocellular carcinoma. Vascular endothelial growth factor (VEGF) is overexpressed in hepatocellular carcinoma (HCC) and interacts with vascular endothelial growth factor receptor (VEGFR) in the vascular endothelium to promote tumor growth. Molecularly targeted therapies focus on VEGF/VEGFR inhibitors, including multi-tyrosine kinase inhibitors (Multi-TKIs) and anti-VEGF can suppress angiogenesis and thus exert an anticancer effect. CD8+ T cells exhibit the expression of immune checkpoint molecules programmed cell death-1 (PD-1), cytotoxic T-lymphocyte antigen 4 (CTLA-4), mucin domain containing-3 (TIM3), and lymphocyte-activation gene 3 (LAG3) on their surface. High expression of CTLA-4 and TIM3 are displayed on the surface of T_regs_. Tumor-associated macrophages (TAMs) and natural killer (NK) cells markedly express TIM3. Binding of PD-1 with its ligand programmed cell death ligand 1 (PD-L1) expressed on tumor cells promotes CD8+ T-cell apoptosis. CTLA-4 inhibits the proliferation of T cells and induces the activity of T_regs_ by binding to CD80/86 in antigen-presenting cells (APCs). The interaction between TIM3 and ligand galectin-9 on the surface of myeloid-derived suppressor cells (MDSCs) also induces T-cell apoptosis. Immune checkpoint inhibitors (ICIs) prevent the inactivation of T cells by blocking the interactions between immune checkpoint molecules with their ligands, thereby exerting antitumor effects.

As shown above, in the TME of HCC, immune checkpoint molecules (PD-1, PD-L1, CTLA4, TIM3, LAG3) are associated with immunosuppressive cells to promote tumor growth and immune escape. This novel finding indicates that there are strong reasons to treat HCC patients with immunotherapies, especially ICI therapy. Increasingly, monoclonal antibodies aimed at blocking these immune checkpoint molecules have attracted increased attention in the HCC landscape (**Figure 2** and **Table 1**) (83).

**Table 1:** Clinical trials with ICIs in HCC.

NCT	Number	Drug Type	Drug	Stage	ORR (%)	DCR (%)	mPFS (months)	mOS (months)	TRAEs(%)	First Posted(year)	Status
Monotherapy											
NCT01658878	48/214	Anti-PD-1	Nivolumab	Phase 1/2	15/20	58/64	3.4/4.0	15.0/NR	25.0	2012	Active, not recruiting
NCT02576509	743	Anti-PD-1	Nivolumab	Phase 3	NA	NA	NA	16.4	49.6	2015	Active, not recruiting
NCT02702414	104	Anti-PD-1	Pembrolizumab	Phase 2	17.0	62.0	4.9	12.9	25.0	2016	Active, not recruiting
NCT02702401	413	Anti-PD-1	Pembrolizumab	Phase 3	18.3	62.2	3 .0	13.9	52.7	2016	Completed
NCT01693562	40	Anti-PD-L1	Durvalumab	Phase 1/2	10.3	33.0	NA	13.2	20.0	2012	Completed
NCT03389126	30	Anti-PD-L1	Avelumab	Phase 2	10.0	73.3	4.4	14.2	19.4	2018	Completed
NCT01008358	21	Anti CTLA-4	Tremelimumab	Phase 2	NA	76.4	6.5	8.2	45	2009	Completed
NCT01853618	32	Anti CTLA-4	Tremelimumab	Phase 1	NA	NA	7.4	12.3	13.0	2013	Completed
ICIs Combinations											
NCT01658878	148	Anti-PD-1 + Anti CTLA-4	Nivolumab + Ipilimumab	Phase 1/2	31.0	49.0	NA	22.8	2.1	2012	Active, not recruiting
NCT03222076	27	Anti-PD-1 + Anti CTLA-4	Nivolumab + Ipilimumab	Phase 2	NA	NA	19.5	NA	43.0%	2017	Active, not recruiting
NCT02519348	332	Anti-PD-L1 + Anti CTLA-4	Durvalumab + Tremelimumab	Phase 1/2	24.0	NA	2.2	18.7	37.8	2015	Active, not recruiting
ICIs combined with Anti-angiogenesis											
NCT03006926	104	Anti-PD-1 + TKIs	Pembrolizumab + Lenvatinib	Phase 1	46.0	NA	9.3	22.0	67.0	2016	Active, not recruiting
NCT03299946	15	Anti-PD-1 + TKIs	Nivolumab + Cabozantinib	Phase 1	NA	NA	NA	NA	NA	2017	Active, not recruiting
NCT03755791	740	Anti-PD-L1 + TKIs	Atezolizumab + Cabozantinib	Phase 3	NA	NA	NA	NA	NA	2018	Recruiting
NCT03794440	595	PD-1 inhibitor + Anti-VEGF	Sintilimab + IBI305	Phase 2/3	NA	NA	4.6	NR	14.0	2019	Active, not recruiting
NCT02715531	223	Anti-PD-L1 + Anti-VEGF	Atezolizumab + Bevacizumab	Phase 1	20.0	NA	5.6	NR	5.0	2016	Completed
NCT03434379	501	Anti-PD-L1 + Anti-VEGF	Atezolizumab + Bevacizumab	Phase 3	27.3	NA	6.8	NR	61.1	2018	Active, not recruiting

ICIs, immune checkpoints inhibitors; ORR, objective response rate; DCR, disease control rate; mPFS, median progression free survival; mOS, median overall survival; TRAEs, treatment-related adverse events; PD-1, programmed cell death-1; PD-L1, programmed cell death ligand 1; CTLA4, cytotoxic T-lymphocyte-associated protein 4; TKIs, tyrosine kinase inhibitors; VEGF, vascular endothelial growth factor; NR, not reached; NA, not available.

### PD-1/PD-L1 Monotherapy

PD-1 is mainly expressed on activated CD4+ and CD8+ T cells and NK cells. PD-L1, the ligand for PD-1, is mainly expressed on APCs and HCC tumor cells. Coinhibitory signals are mediated by the binding of PD-1 and PD-L1 to suppress T-cell immunity. In HCC, it has been shown that the upregulation of PD-1 and PD-L1 induced by various cytokines contributes to the dysfunction of effector T cells, which eventually promotes tumor aggressiveness and recurrence (84, 85). Clinically, the CheckMate-040 study is a multicohort, open label, phase 1/2 trial on the anti-PD-1 antibody nivolumab in patients with advanced HCC. In the dose-escalation phase, a total of 48 advanced HCC patients were enrolled into 3 groups (virus-uninfected, HBV, HCV-infected). The objective response rate (ORR) was 15% (95% CI, 6-28), and the disease control rate (DCR) was 58% (95% CI 43-72). Furthermore, the median progression-free survival (PFS) was 3.4 months (95% CI, 1.6-6.9), and the median overall survival (OS) was 15.0 months (95% CI 9.6-20.2). Severe grade 3/4 treatment-related adverse events (TRAEs), including diarrhea and hepatitis, were observed in 12 (25%) out of 48 patients. In addition, in the dose-expansion phase, a total of 214 advanced HCC patients were enrolled into 4 cohorts, including uninfected sorafenib refractory (n = 57), uninfected sorafenib intolerance (n = 56), HCV infected (n = 50), and HBV infected (n = 51). The ORR was 20% (95% CI 15-26), the DCR was reported as 64% (95% CI, 50-71), and the median PFS was 4.0 months (95% CI, 2.9-5.4). The OS was not reached. Nivolumab may offer favorable efficacy with a manageable safety profile, and a phase 3 randomized trial compared with sorafenib is underway (86). In another phase 3 trial (CheckMate-459) 743 systemic therapy-naive patients with advanced HCC were recruited to verify the effects of nivolumab compared with sorafenib. The median OS was 16.4 months (95% CI, 13.9-18.4) for nivolumab and 14.7 months (95% CI, 11.9-17.2) for sorafenib. TRAEs were reported in 82 patients (22.3%) and 180 patients (49.6%) treated with nivolumab and sorafenib, respectively (87). In KEYNOTE-224, a phase 2 trial, the efficacy and safety of pembrolizumab (anti-PD-1 antibody) were evaluated in 104 HCC patients who had progressed or were intolerant to sorafenib. The ORR was recorded as 17% (95% CI, 11-26), and the DCR was 62% (95% CI, 52-71). The median PFS and OS were 4.9 months (95% CI, 3.4-7.2) and 12.9 months (95% CI, 9.7-15.5), respectively. Twenty-six (25%) grade 3-4 TRAEs were observed (88). In addition, in a randomized, multicenter phase 3 trial (KEYNOTE-240) the efficacy and safety of pembrolizumab compared with a placebo were assessed in 413 HCC patients after progression on sorafenib. The results indicated that the ORR and DCR of the pembrolizumab group were 18.3% (95% CI, 14.0-23.4) and 62.2%, respectively, which was significantly better than those of the control group (4.4% (95% CI, 1.6-9.4) and 53.3%, respectively. The median PFS and OS for pembrolizumab were 3.0 months (95% CI, 2.8-4.1) and 13.9 months (95% CI, 11.6-16.0) versus 2.8 months (95% CI, 1.6-3.0) and 10.6 months (95% CI, 8.3-13.5) for the placebo, respectively. The incidence of TRAEs of grade 3 and above was 52.7% in the pembrolizumab group and 46.3% in the placebo group. Anti-PD-L1 monotherapy (durvalumab) was observed as part of a randomized expansion phase 1/2 study in 104 HCC patients who progressed or refused sorafenib treatment, with an ORR of 10.6% (95% CI, 5.4-18.1) and a median OS of 13.6 months (8.7 to 17.6) (89). At the 2017 American Society of Clinical Oncology (ASCO) annual meeting, an ongoing phase 1/2 trial that was aimed at evaluating the safety and clinical activity of durvalumab in advanced solid tumors showed promising antitumor activity and management safety in 40 patients with HCC (5). The fully human monoclonal anti-PD-L1 agent avelumab underwent further assessment in a phase 2, single-arm, single center in patients with advanced HCC who were previously treated with sorafenib (NCT03389126). Preliminary results were promising: ORR: 10.0%, DCR: 73.3%, median PFS: 4.4 months (95% CI, 2.9-5.9), median OS: 14.2 months (95% CI, 9.5-18.9). Avelumab was well tolerated with manageable toxicity, with 7 grade 3 TRAEs and no grade 4 TRAEs (90).

### CTLA-4 Monotherapy

CTLA-4 is present on T_regs_ and activated T cells and is an inhibitory coreceptor that plays an important role in regulating the function of CD4+ T cells. In many types of solid cancer, including HCC, CTLA-4 suppresses the proliferation of T cells, promotes the production of the suppressive cytokines IL-10 and IDO, and induces T_reg_ activity (91, 92). Many clinical trials on anti-CTLA-4 are currently ongoing with promising results. The antitumor effect of blocking CTLA-4 with tremelimumab in the treatment of HCV-associated advanced HCC was demonstrated in a phase 2 trial (NCT01008358), involving 21 patients. A remarkable DCR of 76.4% was observed, and although there was no complete response, the partial response rate (PRR) was 17.6%. In the study, the efficacy of tremelimumab was investigated with a median PFS of 6.48 months (95% CI, 3.95-9.14) and a median OS of 8.2 months (95% CI, 4.64-21.34) and a manageable safety profile. In addition, tremelimumab has been shown to play an antiviral role, and a progressive course of decreased viral load was observed for almost 3 months in most patients (93). In another recent communication, a phase 1 trial (NCT01853618) was reported, evaluating tremelimumab in combination with ablation in 32 patients with advanced HCC. The study showed significant results, with a median PFS and OS of 7.4 months (95% CI, 4.7-19.4) and 12.3 (95% CI, 9.3-15.4), respectively. Four (13%) patients presented with grade 3/4 TRAEs (94).

### ICI Combination

Most ICI combination trials in advanced HCC have previously shown efficacy. The combination of the anti-PD-1 antibody nivolumab and the anti-CTLA-4 antibody ipilimumab was first tested in the phase 1/2 CheckMate-040 trial (NCT01658878). Based on different dosages, 148 advanced HCC patients who were previously treated with sorafenib were randomized into three arms: (A) nivolumab 1 mg/kg + ipilimumab 3 mg/kg, (B) nivolumab 3 mg/kg + ipilimumab 1 mg/kg every 3 weeks (Q3 W), and (C) nivolumab 3 mg/kg + ipilimumab 1 mg/kg every 6 weeks (Q6 W). The primary endpoint ORR was 31.0% (95% CI, 18-45) in combination therapy compared to 15% (95% CI, 6-28) in nivolumab monotherapy. At 24 months, the DCR was 48.8%, and the OS was 40%. A promising effect on outcome was observed, especially in arm A, with a median OS of 22.8 months (95% CI 9.4 - not reached). Grade 3-4 TRAEs were reported in 5 out of 49 patients (10.2%) in arm A, 2 out of 49 patients (4.1%) in arm B, and 1 out of 48 patients (2.1%) in arm C (95). Based on these promising results, in March 2020, the FDA approved combination therapy (arm A) as a second-line treatment after sorafenib. Recently, an open-label, randomized phase 2 trial (NCT03222076) evaluated the efficacy of nivolumab monotherapy versus nivolumab plus ipilimumab in the treatment of HCC patients who could be treated by surgery. All 27 patients were classified into nivolumab monotherapy (n = 13) and nivolumab plus ipilimumab combination therapy (n = 14) groups. Feasible data were observed, with a median PFS of 19.53 months (95% CI, 2.33 - not estimable) in the combination group and 9.4 months (95% CI, 1.47 - not estimable) in the monotherapy group. However, in combination therapy, grade 3-4 TRAEs (6 of 14 (43.0%)) were higher than those of nivolumab alone (3 of 13 (23.0%)). Overall, nivolumab plus ipilimumab appeared to be safe and effective (96). Clinical data on durvalumab (anti-PD-L1) in combination with tremelimumab (anti-CTLA-4) were presented in a phase 1/2 study including 332 HCC patients. Four cohorts were assigned, including T300 + D (tremelimumab 300 mg + durvalumab), durvalumab or tremelimumab monotherapy, and T75 + D (tremelimumab 75 mg + durvalumab). The results showed that the ORR and median OS of the T300 + D cohort were 24.0% (95% CI, 14.9-35.3) and 18.7 months (95% CI, 10.8-27.3), respectively, which were better than the data obtained from monotherapy and T75 + D groups. However, the incidence of grade ≥ 3 TRAEs was the highest (37.8%) in the 4 groups (86). Recently, TIM3 has been shown to overcome resistance to PD-1 blockade (97). The results for a phase 2 trial assessing the efficacy and safety of anti-PD-1 and anti-TIM3 combination therapy (NCT03680508) (98) are still awaited. In addition, dual blockade of PD-1 with anti-LAG3 therapy is being conducted in a phase 1 trial (NCT01968109). However, the clinical values of TIM3 and LAG3 need to be further elucidated.

### ICIs Combined With Anti-Angiogenesis

Additional strategies aimed at combining TKIs/anti-VEGFs with ICI therapy after ICI progression may represent future treatment options. A total of 104 patients were enrolled in a phase 1b, multicenter, open-label trial of lenvatinib (TKIs) plus pembrolizumab (anti-PD-1) in patients with unresectable HCC. Patients received lenvatinib (12 mg if ≥ 60 kg, 8 mg if < 60 kg) orally daily plus pembrolizumab 200 mg Q3 W intravenously on day 1 of a 3-week cycle. The ORR was 46.0% (95% CI, 36.0-56.3), with a median PFS of 9.3 months and a median OS of 22.0 months. Grade ≥3 TRAEs occurred in 67% of patients, and no new safety signals were observed (99). A cohort study was launched within the single arm phase 1b trial (NCT03299946) exploring the combination of cabozantinib (TKIs) and nivolumab (anti-PD-1) in locally advanced HCC patients. A total of 15 patients were included in the study; 12 out of 15 patients (80%) underwent surgical resection after combination therapy, and 5 out of 15 patients (42%) had major pathologic responses (100). A COSMIC-312 phase 3 study trial of cabozantinib in combination with atezolizumab (anti-PD-L1) versus sorafenib is currently ongoing (NCT03755791) in treatment-naive HCC patients. Approximately 740 patients were randomized into 3 groups: cabozantinib plus atezolizumab (370 patients) and sorafenib or cabozantinib single-agent (185 patients). For the combination group, cabozantinib was administered orally (40 mg once daily) plus atezolizumab 1200 mg Q3 W intravenously (101). Currently, clinical trials are ongoing. Recently, an open-label, phase 2-3 trial (NCT03794440) was performed in 595 unresectable HBV-associated HCC patients in China. First, a phase 2 study was performed in 24 patients, and inspiring results were obtained. The ORR in the phase 2 part of the study was 25.0% (95% CI, 9.8-46.7), and TRAEs were observed in 7 out of 24 patients (29%). Subsequently, a randomized phase 3 trial was started because of its preliminary safety profile and effectiveness. The remaining 571 patients were randomly assigned to the sintilimab (PD-1 inhibitor) plus IBI305 (anti-VEGF agent bevacizumab biosimilar) group (n = 380) or sorafenib group (n = 191). This trial demonstrated that patients with sintilimab plus IBI305 combination treatment had a significantly longer median PFS (4.6 months (95% CI, 4.1-5.7)) and median OS (not reached) than patients in the sorafenib group (median PFS and OS were 2.8 months and 10.4 months, respectively) (102). In GO30140, an open-label, multicenter, phase 1b trial (NCT02715531), two unresectable HCC cohorts, groups A and F, from 26 academic centers were described. In group A, 104 patients were enrolled and treated with atezolizumab plus bevacizumab (anti-VEGF). In group F, 119 patients were enrolled and randomly assigned into 2 groups: atezolizumab combined with bevacizumab (n = 60) and atezolizumab monotherapy (n = 59). In group A and in the combination therapy subgroup in group F, all patients received 1200 mg atezolizumab and 15 mg/kg bevacizumab intravenously Q3 W. P patients in the other group of group F were given only 1200 mg atezolizumab intravenously Q3 W. The results showed that the ORR (20%, (95% CI, 11-32)) and median PFS (5.6 (95% CI, 3.6-7.4)) of patients in the atezolizumab plus bevacizumab group were superior to those of patients who received atezolizumab monotherapy (103). A total of 501 unresectable HCC patients who had not previously received systemic treatment were enrolled in a global, phase 3 clinical trial (IMbrave150, NCT03434379) and were randomly divided in a 2:1 ratio into two groups: atezolizumab plus bevacizumab therapy (336 patients) or sorafenib therapy (165 patients). Patients in the combined therapy arm were treated with a standard dose (1200 mg) of atezolizumab followed by a high dose of bevacizumab (15 mg/kg) Q3 W, and patients in the sorafenib arm orally received 400 mg twice daily. After treatment, according to the RECIST 1.1 criteria, we showed that the ORR was 27.3% (95% CI, 22.5-32.5) in the atezolizumab plus bevacizumab group and 11.9% (95% CI, 7.4-18.0) in the sorafenib group. A prognostic advantage of combination therapy over sorafenib was also observed. The median PFS was 6.8 months (95% CI, 5.7-8.3), which was significantly longer than the 4.3 months (95% CI, 4.0-5.6) in the sorafenib group. The median OS was 13.2 months (95% CI, 10.4 - not reached) with sorafenib but was not reached in the combined group. For safety, 201 patients (61.1%) with serious TRAEs (≥ grade 3) were observed in the atezolizumab plus bevacizumab arm, and 95 patients (60.9%) were observed in the sorafenib arm (104).

## Challenges in Combination Therapy for HCC

Despite encouraging preliminary data generated using combination strategies of antiangiogenic therapy and ICIs of advanced HCC, challenges still represent a burden in HCC management.

### Drug Resistance of Combination Therapy

One of the main challenges is drug resistance (primary or acquired), which remains the major cause of treatment failure. Drug resistance is complex and dynamic because abnormal behavior at any step can lead to drug resistance. In recent years, various molecular mechanisms underlying drug resistance have been investigated and identified (105). First, HCC is generally considered an “immune-cold” tumor, characterized by T-cell deficiency, infiltration of immunosuppressive cells (MDSCs, TAMs, T_regs_) and poor antigen presentation, resulting in the ability to maintain immune tolerance and an inability to produce tumor immune responses (106). The characteristic of a “cold” HCC tumor is the common mechanism for primary resistance (107). Moreover, tumor heterogeneity is the other underlying mechanism involved in primary resistance. Unlike other primary tumors, multifocal lesions in the liver are common and although these tumors genetically originate from similar cells, they differ significantly from each other. It is not only the multifocal tumors that cause the heterogeneity of HCC but also the difference in patients for the differential expression of immune checkpoint molecules (108). Thus, it is critical to develop new ways to diminish primary drug resistance by transforming the “cold” tumor microenvironment into a “hot” tumor as well as circumventing tumor heterogeneity. In addition, the heterogeneity of the HCC TME also plays an important role in later acquired resistance. In previous studies, it has been shown that approximately a quarter of HCC (classified as immune class) has higher immune infiltration and higher PD-1/PD-L1 expression levels and thus has higher response rates to immunotherapy than the rest of HCCs (109, 110). However, a high response to treatment cannot be guaranteed for immune-suppressive cells, including MDSCs, TAMs, and T_regs_, in the TME of HCC. These immune-suppressive components of the TME may contribute to T-cell exhaustion and immune checkpoint protein dysfunction, which further develop drug resistance to ICIs. Thus, a model that stratifies HCC patients according to the status of immune infiltration and immune checkpoint molecules may help to adequately select candidates for ICI therapy (111).

Intratumor heterogeneity is the key reason for sorafenib therapy resistance. In a previous study, it was demonstrated that sorafenib can induce the accumulation of autophagosomes in an *in vitro* HCC model. Several studies have reported that sorafenib induces an autophagic-protective response in HCC cells, resulting in drug resistance and affecting therapeutic efficacy. In addition, an imbalance between anti-apoptotic and pro-apoptotic proteins is associated with sorafenib resistance. Nonetheless, the exact underlying mechanism of sorafenib resistance needs to be further elucidated (112).

In the author’s opinion, physicians need to consider individual differences in the treatment process and specify individual diagnosis and treatment plans to improve treatment efficacy. In addition, further studies on HCC immunity and molecular pathology are needed to elucidate the underlying mechanisms involved in the TME that may lead to the failure of immunotherapy.

### Potential Biomarkers of Clinical Response in Combination Therapy

The potential of ICIs in combination with TKIs/anti-VEGFs for HCC has been widely recognized. Moreover, many clinical trials have indicated that not all HCC patients receiving combination treatment will achieve the desired efficacy. Biomarkers are good indicators for predicting and evaluating treatment response. Recently, a meta-analysis indicated that patients with high PD-L1 expression (>1% score) had longer survival than patients with <1% PD-L1 expression. Therefore, the PD-L1 status may be a potential predictive biomarker in the context of anti-PD-1/PD-L1 therapy (98). Moreover, one study showed that HCC patients with Wnt/CTNNB1 mutations were insensitive to anti-PD-1/PD-L1 therapy and had a worse prognosis than patients without mutations (113). Furthermore, relevant literature shows that CD28/B7 may be a biomarker for the clinical response to anti-PD-1 in mouse models and lung cancer patients. However, clinical data from HCC patients are insufficient (114). In another preclinical study, it was suggested that HCC patients with high AFP levels (≥400 ng/mL) are more likely to profit from combination therapy of ICIs and lenvatinib (115). Accordingly, there is a need to develop predictive biomarkers with high specificity and sensitivity to accurately identify HCC patients who most likely benefit from combination therapy. As mentioned above, comprehensive and systematic studies on the molecular level of HCC immunity are warranted.

### TRAEs of Combination Therapy

TRAEs are one of the most concerning issues in clinical trials and not only affect the quality of life of patients but also affect treatment compliance. TRAEs are classified into five grades based on severity, and TRAEs of grade 3 or more are considered serious TRAEs. The most common TRAEs occur in the skin, gastrointestinal, liver, lung, and endocrine systems (116). Skin toxicity and gastrointestinal toxicity (diarrhea and colitis) were the first and second most common TRAEs, respectively. The incidence of skin and gastrointestinal toxicity in patients who were treated with anti-PD-1/PD-L1 is approximately 30.0% and 10.0-20.0%, respectively. For anti-CTLA-4 treatment, skin and gastrointestinal toxicity was as high as 40.0% and 30.0-40.0%, respectively. It has been suggested that anti-PD1/PDL1 results in fewer TRAEs than anti-CTLA-4. Moreover, a combination of anti-PD1 with anti-CTLA-4 showed an increased hepatic TRAEs in the early phase of treatment, although most improved after six weeks (117). Of note, fatal cardiotoxicity has been reported in patients who were treated with pembrolizumab or a combination of nivolumab and ipilimumab. Most TRAEs are reversible and controllable, but severe cardiac and autoimmune diseases should receive more attention for early recognition and intervention in the future (118).

In the author’s opinion, the proportion of patients with advanced HCC complicated with HBV in China is high, and the associated TRAEs are more complex. Therefore, no matter which treatment method is chosen, special attention should be paid to a patients’ underlying liver disease and other underlying diseases.

## Conclusion

More than 70% of HCC patients are diagnosed at an intermediate or advanced stage (BCLC stage B, C or D) and require systemic therapy. The clinical efficacy of traditional TKI drugs (sorafenib, lenvatinib etc.) is still not satisfactory, although once brought patients hope. Thus, novel strategies are currently being developed, including ICIs, ICI combinations, and ICI combinations with antiangiogenics. More recently, the application of ICI-based combination therapy has become a growing field of study that has gradually displaced TKI monotherapy in advanced HCC. However, challenges remain, including drug resistance, predictive biomarkers of treatment effectiveness, and TRAEs in combination treatment. More safe and effective combination therapy strategies for advanced HCC should be developed, and further studies are needed.

## Author Contributions

CZ contributed to the study design. YC and HH were responsible for data collection and prepared the manuscript. XY and XF drafted and prepared the manuscript. All authors participated in the data interpretation, contributed to the manuscript writing with important intellectual content and approved the final version of the manuscript.

## Conflict of Interest

The authors declare that the research was conducted in the absence of any commercial or financial relationships that could be construed as a potential conflict of interest.

## Publisher’s Note

All claims expressed in this article are solely those of the authors and do not necessarily represent those of their affiliated organizations, or those of the publisher, the editors and the reviewers. Any product that may be evaluated in this article, or claim that may be made by its manufacturer, is not guaranteed or endorsed by the publisher.

## References

[1] Sung H Ferlay J Siegel RL Laversanne M Soerjomataram I Jemal A. Global Cancer Statistics 2020: Globocan Estimates of Incidence and Mortality Worldwide for 36 Cancers in 185 Countries. CA Cancer J Clin (2021) 71(3):209–49. doi: 10.3322/caac.21660 33538338

[2] Ogasawara S Choo SP Li JT Yoo C Wang B Lee D. Evolving Treatment of Advanced Hepatocellular Carcinoma in the Asia-Pacific Region: A Review and Multidisciplinary Expert Opinion. Cancers (Basel) (2021) 13(11):2626. doi: 10.3390/cancers13112626 34071818 PMC8197840

[3] Sangro B Sarobe P Hervas-Stubbs S Melero I. Advances in Immunotherapy for Hepatocellular Carcinoma. Nat Rev Gastroenterol Hepatol (2021) 18(8):525–43. doi: 10.1038/s41575-021-00438-0 PMC8042636 33850328

[4] Yang JD Hainaut P Gores GJ Amadou A Plymoth A Roberts LR. A Global View of Hepatocellular Carcinoma: Trends, Risk, Prevention and Management. Nat Rev Gastroenterol Hepatol (2019) 16(10):589–604. doi: 10.1038/s41575-019-0186-y 31439937 PMC6813818

[5] Hilmi M Neuzillet C Calderaro J Lafdil F Pawlotsky JM Rousseau B. Angiogenesis and Immune Checkpoint Inhibitors as Therapies for Hepatocellular Carcinoma: Current Knowledge and Future Research Directions. J Immunother Cancer (2019) 7(1):333. doi: 10.1186/s40425-019-0824-5 31783782 PMC6884868

[6] Forner A Reig M Bruix J. Hepatocellular Carcinoma. Lancet (2018) 391(10127):1301–14. doi: 10.1016/s0140-6736(18)30010-2 29307467

[7] European Association for the Study of the Liver European Association for the Study of the L. Easl Clinical Practice Guidelines: Management of Hepatocellular Carcinoma. J Hepatol (2018) 69(1):182–236. doi: 10.1016/j.jhep.2018.03.019 29628281

[8] Tsochatzis EA Bosch J Burroughs AK. Liver Cirrhosis. Lancet (2014) 383(9930):1749–61. doi: 10.1016/S0140-6736(14)60121-5 24480518

[9] Morse MA Sun W Kim R He AR Abada PB Mynderse M. The Role of Angiogenesis in Hepatocellular Carcinoma. Clin Cancer Res (2019) 25(3):912–20. doi: 10.1158/1078-0432.CCR-18-1254 30274981

[10] Liu Z Lin Y Zhang J Zhang Y Li Y Liu Z. Molecular Targeted and Immune Checkpoint Therapy for Advanced Hepatocellular Carcinoma. J Exp Clin Cancer Res (2019) 38(1):447. doi: 10.1186/s13046-019-1412-8 31684985 PMC6827249

[11] Llovet JM Ricci S Mazzaferro V Hilgard P Gane E Blanc JF. Sorafenib in Advanced Hepatocellular Carcinoma. N Engl J Med (2008) 359(4):378–90. doi: 10.1056/NEJMoa0708857 18650514

[12] Cheng AL Kang YK Chen Z Tsao CJ Qin S Kim JS. Efficacy and Safety of Sorafenib in Patients in the Asia-Pacific Region With Advanced Hepatocellular Carcinoma: A Phase Iii Randomised, Double-Blind, Placebo-Controlled Trial. Lancet Oncol (2009) 10(1):25–34. doi: 10.1016/S1470-2045(08)70285-7 19095497

[13] Abdelrahim M Esmail A Abudayyeh A Murakami N Saharia A McMillan R. Transplant Oncology: An Evolving Field in Cancer Care. Cancers (Basel) (2021) 13(19):4911. doi: 10.3390/cancers13194911 34638395 PMC8508383

[14] Abdelrahim M Victor D Esmail A Kodali S Graviss EA Nguyen DT. Transarterial Chemoembolization (Tace) Plus Sorafenib Compared to Tace Alone in Transplant Recipients With Hepatocellular Carcinoma: An Institution Experience. Cancers (Basel) (2022) 14(3):650. doi: 10.3390/cancers14030650 35158918 PMC8833802

[15] Benson AB D'Angelica MI Abbott DE Abrams TA Alberts SR Anaya DA. Guidelines Insights: Hepatobiliary Cancers, Version 2.2019. J Natl Compr Canc Netw (2019) 17(4):302–10. doi: 10.6004/jnccn.2019.0019 30959462

[16] Benson AB 3rd D'Angelica MI Abbott DE Abrams TA Alberts SR Saenz DA. Nccn Guidelines Insights: Hepatobiliary Cancers, Version 1.2017. J Natl Compr Canc Netw (2017) 15(5):563–73. doi: 10.6004/jnccn.2017.0059 PMC5557008 28476736

[17] Zhu AX Kang YK Yen CJ Finn RS Galle PR Llovet JM. Ramucirumab After Sorafenib in Patients With Advanced Hepatocellular Carcinoma and Increased Alpha-Fetoprotein Concentrations (Reach-2): A Randomised, Double-Blind, Placebo-Controlled, Phase 3 Trial. Lancet Oncol (2019) 20(2):282–96. doi: 10.1016/S1470-2045(18)30937-9 30665869

[18] Sharma R Motedayen Aval L. Beyond First-Line Immune Checkpoint Inhibitor Therapy in Patients With Hepatocellular Carcinoma. Front Immunol (2021) 12:652007. doi: 10.3389/fimmu.2021.652007 33790915 PMC8005707

[19] Zongyi Y Xiaowu L. Immunotherapy for Hepatocellular Carcinoma. Cancer Lett (2020) 470:8–17. doi: 10.1016/j.canlet.2019.12.002 31811905

[20] Zhou G Boor PPC Bruno MJ Sprengers D Kwekkeboom J. Immune Suppressive Checkpoint Interactions in the Tumour Microenvironment of Primary Liver Cancers. Br J Cancer (2022) 126(1):10–23. doi: 10.1038/s41416-021-01453-3 34400801 PMC8727557

[21] He X Xu C. Immune Checkpoint Signaling and Cancer Immunotherapy. Cell Res (2020) 30(8):660–9. doi: 10.1038/s41422-020-0343-4 PMC7395714 32467592

[22] Liu ZL Liu JH Staiculescu D Chen J. Combination of Molecularly Targeted Therapies and Immune Checkpoint Inhibitors in the New Era of Unresectable Hepatocellular Carcinoma Treatment. Ther Adv Med Oncol (2021) 13:17588359211018026. doi: 10.1177/17588359211018026 34104226 PMC8150670

[23] Abdelrahim M Esmail A Saharia A Abudayyeh A Abdel-Wahab N Diab A. Utilization of Immunotherapy for the Treatment of Hepatocellular Carcinoma in the Peri-Transplant Setting: Transplant Oncology View. Cancers (Basel) (2022) 14(7):1760. doi: 10.3390/cancers14071760 35406533 PMC8997123

[24] Dong Y Wong JSL Sugimura R Lam KO Li B Kwok GGW. Recent Advances and Future Prospects in Immune Checkpoint (Ici)-Based Combination Therapy for Advanced Hcc. Cancers (Basel) (2021) 13(8):1949. doi: 10.3390/cancers13081949 33919570 PMC8072916

[25] Rizzo A Ricci AD Gadaleta-Caldarola G Brandi G. First-Line Immune Checkpoint Inhibitor-Based Combinations in Unresectable Hepatocellular Carcinoma: Current Management and Future Challenges. Expert Rev Gastroenterol Hepatol (2021) 15(11):1245–51. doi: 10.1080/17474124.2021.1973431 34431725

[26] Johnston MP Khakoo SI. Immunotherapy for Hepatocellular Carcinoma: Current and Future. World J Gastroenterol (2019) 25(24):2977–89. doi: 10.3748/wjg.v25.i24.2977 PMC6603808 31293335

[27] Leone P Solimando AG Fasano R Argentiero A Malerba E Buonavoglia A. The Evolving Role of Immune Checkpoint Inhibitors in Hepatocellular Carcinoma Treatment. Vaccines (Basel) (2021) 9(5):532. doi: 10.3390/vaccines9050532 34065489 PMC8160723

[28] El Dika I Khalil DN Abou-Alfa GK. Immune Checkpoint Inhibitors for Hepatocellular Carcinoma. Cancer (2019) 125(19):3312–9. doi: 10.1002/cncr.32076 PMC7944520 31290997

[29] Thomson AW Knolle PA. Antigen-Presenting Cell Function in the Tolerogenic Liver Environment. Nat Rev Immunol (2010) 10(11):753–66. doi: 10.1038/nri2858 20972472

[30] Elsegood CL Tirnitz-Parker JE Olynyk JK Yeoh GC. Immune Checkpoint Inhibition: Prospects for Prevention and Therapy of Hepatocellular Carcinoma. Clin Transl Immunol (2017) 6(11):e161. doi: 10.1038/cti.2017.47 PMC5704099 29326816

[31] Guardascione M Toffoli G. Immune Checkpoint Inhibitors as Monotherapy or Within a Combinatorial Strategy in Advanced Hepatocellular Carcinoma. Int J Mol Sci (2020) 21(17):6302. doi: 10.3390/ijms21176302 PMC7504231 32878115

[32] Pardoll DM. The Blockade of Immune Checkpoints in Cancer Immunotherapy. Nat Rev Cancer (2012) 12(4):252–64. doi: 10.1038/nrc3239 PMC4856023 22437870

[33] Li B Yan C Zhu J Chen X Fu Q Zhang H. Anti-Pd-1/Pd-L1 Blockade Immunotherapy Employed in Treating Hepatitis B Virus Infection-Related Advanced Hepatocellular Carcinoma: A Literature Review. Front Immunol (2020) 11:1037. doi: 10.3389/fimmu.2020.01037 32547550 PMC7270402

[34] Hu H Chen Y Tan S Wu S Huang Y Fu S. The Research Progress of Antiangiogenic Therapy, Immune Therapy and Tumor Microenvironment. Front Immunol (2022) 13:802846. doi: 10.3389/fimmu.2022.802846 35281003 PMC8905241

[35] Giraud J Chalopin D Blanc JF Saleh M. Hepatocellular Carcinoma Immune Landscape and the Potential of Immunotherapies. Front Immunol (2021) 12:655697. doi: 10.3389/fimmu.2021.655697 33815418 PMC8012774

[36] Zheng X Xu M Yao B Wang C Jia Y Liu Q. Il-6/Stat3 Axis Initiated Cafs *Via* Up-Regulating Timp-1 Which Was Attenuated by Acetylation of Stat3 Induced by Pcaf in Hcc Microenvironment. Cell Signal (2016) 28(9):1314–24. doi: 10.1016/j.cellsig.2016.06.009 27297362

[37] Yin Z Ma T Lin Y Lu X Zhang C Chen S. Il-6/Stat3 Pathway Intermediates M1/M2 Macrophage Polarization During the Development of Hepatocellular Carcinoma. J Cell Biochem (2018) 119(11):9419–32. doi: 10.1002/jcb.27259 30015355

[38] Yang R Gao N Chang Q Meng X Wang W. The Role of Ido, Il-10, and Tgf-Beta in the Hcv-Associated Chronic Hepatitis, Liver Cirrhosis, and Hepatocellular Carcinoma. J Med Virol (2019) 91(2):265–71. doi: 10.1002/jmv.25083 29611873

[39] Shen X Zhang L Li J Li Y Wang Y Xu ZX. Recent Findings in the Regulation of Programmed Death Ligand 1 Expression. Front Immunol (2019) 10:1337. doi: 10.3389/fimmu.2019.01337 31258527 PMC6587331

[40] Liu HT Jiang MJ Deng ZJ Li L Huang JL Liu ZX. Immune Checkpoint Inhibitors in Hepatocellular Carcinoma: Current Progresses and Challenges. Front Oncol (2021) 11:737497. doi: 10.3389/fonc.2021.737497 34745958 PMC8570111

[41] Chen KJ Lin SZ Zhou L Xie HY Zhou WH Taki-Eldin A. Selective Recruitment of Regulatory T Cell Through Ccr6-Ccl20 in Hepatocellular Carcinoma Fosters Tumor Progression and Predicts Poor Prognosis. PloS One (2011) 6(9):e24671. doi: 10.1371/journal.pone.0024671 21935436 PMC3173477

[42] Yamane H Paul WE. Early Signaling Events That Underlie Fate Decisions of Naive Cd4(+) T Cells Toward Distinct T-Helper Cell Subsets. Immunol Rev (2013) 252(1):12–23. doi: 10.1111/imr.12032 23405892 PMC3578301

[43] Zou W. Regulatory T Cells, Tumour Immunity and Immunotherapy. Nat Rev Immunol (2006) 6(4):295–307. doi: 10.1038/nri1806 16557261

[44] Yuan CH Sun XM Zhu CL Liu SP Wu L Chen H. Amphiregulin Activates Regulatory T Lymphocytes and Suppresses Cd8+ T Cell-Mediated Anti-Tumor Response in Hepatocellular Carcinoma Cells. Oncotarget (2015) 6(31):32138–53. doi: 10.18632/oncotarget.5171 PMC4741664 26451607

[45] Huang Y Wang FM Wang T Wang YJ Zhu ZY Gao YT. Tumor-Infiltrating Foxp3+ Tregs and Cd8+ T Cells Affect the Prognosis of Hepatocellular Carcinoma Patients. Digestion (2012) 86(4):329–37. doi: 10.1159/000342801 23207161

[46] Shevach EM. Mechanisms of Foxp3+ T Regulatory Cell-Mediated Suppression. Immunity (2009) 30(5):636–45. doi: 10.1016/j.immuni.2009.04.010 19464986

[47] Larmonier N Marron M Zeng Y Cantrell J Romanoski A Sepassi M. Tumor-Derived Cd4(+)Cd25(+) Regulatory T Cell Suppression of Dendritic Cell Function Involves Tgf-Beta and Il-10. Cancer Immunol Immunother (2007) 56(1):48–59. doi: 10.1007/s00262-006-0160-8 16612596 PMC11030031

[48] Oura K Morishita A Tani J Masaki T. Tumor Immune Microenvironment and Immunosuppressive Therapy in Hepatocellular Carcinoma: A Review. Int J Mol Sci (2021) 22(11):5801. doi: 10.3390/ijms22115801 34071550 PMC8198390

[49] Deng Y Cheng J Fu B Liu W Chen G Zhang Q. Hepatic Carcinoma-Associated Fibroblasts Enhance Immune Suppression by Facilitating the Generation of Myeloid-Derived Suppressor Cells. Oncogene (2017) 36(8):1090–101. doi: 10.1038/onc.2016.273 27593937

[50] Zhou J Liu M Sun H Feng Y Xu L Chan AWH. Hepatoma-Intrinsic Ccrk Inhibition Diminishes Myeloid-Derived Suppressor Cell Immunosuppression and Enhances Immune-Checkpoint Blockade Efficacy. Gut (2018) 67(5):931–44. doi: 10.1136/gutjnl-2017-314032 PMC5961939 28939663

[51] Chiu DK Xu IM Lai RK Tse AP Wei LL Koh HY. Hypoxia Induces Myeloid-Derived Suppressor Cell Recruitment to Hepatocellular Carcinoma Through Chemokine (C-C Motif) Ligand 26. Hepatology (2016) 64(3):797–813. doi: 10.1002/hep.28655 27228567

[52] Nan J Xing YF Hu B Tang JX Dong HM He YM. Endoplasmic Reticulum Stress Induced Lox-1(+ ) Cd15(+) Polymorphonuclear Myeloid-Derived Suppressor Cells in Hepatocellular Carcinoma. Immunology (2018) 154(1):144–55. doi: 10.1111/imm.12876 PMC5904716 29211299

[53] Kondo Y Shimosegawa T. Significant Roles of Regulatory T Cells and Myeloid Derived Suppressor Cells in Hepatitis B Virus Persistent Infection and Hepatitis B Virus-Related Hccs. Int J Mol Sci (2015) 16(2):3307–22. doi: 10.3390/ijms16023307 PMC4346897 25654227

[54] Fu Y Liu S Zeng S Shen H. From Bench to Bed: The Tumor Immune Microenvironment and Current Immunotherapeutic Strategies for Hepatocellular Carcinoma. J Exp Clin Cancer Res (2019) 38(1):396. doi: 10.1186/s13046-019-1396-4 31500650 PMC6734524

[55] Hoechst B Voigtlaender T Ormandy L Gamrekelashvili J Zhao F Wedemeyer H. Myeloid Derived Suppressor Cells Inhibit Natural Killer Cells in Patients With Hepatocellular Carcinoma *Via* the Nkp30 Receptor. Hepatology (2009) 50(3):799–807. doi: 10.1002/hep.23054 19551844 PMC6357774

[56] Kuang DM Zhao Q Peng C Xu J Zhang JP Wu C. Activated Monocytes in Peritumoral Stroma of Hepatocellular Carcinoma Foster Immune Privilege and Disease Progression Through Pd-L1. J Exp Med (2009) 206(6):1327–37. doi: 10.1084/jem.20082173 PMC2715058 19451266

[57] Noy R Pollard JW. Tumor-Associated Macrophages: From Mechanisms to Therapy. Immunity (2014) 41(1):49–61. doi: 10.1016/j.immuni.2014.06.010 25035953 PMC4137410

[58] Qian BZ Pollard JW. Macrophage Diversity Enhances Tumor Progression and Metastasis. Cell (2010) 141(1):39–51. doi: 10.1016/j.cell.2010.03.014 20371344 PMC4994190

[59] Wan S Kuo N Kryczek I Zou W Welling TH. Myeloid Cells in Hepatocellular Carcinoma. Hepatology (2015) 62(4):1304–12. doi: 10.1002/hep.27867 PMC4589430 25914264

[60] Kuang DM Peng C Zhao Q Wu Y Chen MS Zheng L. Activated Monocytes in Peritumoral Stroma of Hepatocellular Carcinoma Promote Expansion of Memory T Helper 17 Cells. Hepatology (2010) 51(1):154–64. doi: 10.1002/hep.23291 19902483

[61] Yan W Liu X Ma H Zhang H Song X Gao L. Tim-3 Fosters Hcc Development by Enhancing Tgf-Beta-Mediated Alternative Activation of Macrophages. Gut (2015) 64(10):1593–604. doi: 10.1136/gutjnl-2014-307671 25608525

[62] Ye LY Chen W Bai XL Xu XY Zhang Q Xia XF. Hypoxia-Induced Epithelial-To-Mesenchymal Transition in Hepatocellular Carcinoma Induces an Immunosuppressive Tumor Microenvironment to Promote Metastasis. Cancer Res (2016) 76(4):818–30. doi: 10.1158/0008-5472.CAN-15-0977 26837767

[63] Guidotti LG Inverso D Sironi L Di Lucia P Fioravanti J Ganzer L. Immunosurveillance of the Liver by Intravascular Effector Cd8(+) T Cells. Cell (2015) 161(3):486–500. doi: 10.1016/j.cell.2015.03.005 25892224 PMC11630812

[64] Wang X He Q Shen H Xia A Tian W Yu W. Tox Promotes the Exhaustion of Antitumor Cd8(+) T Cells by Preventing Pd1 Degradation in Hepatocellular Carcinoma. J Hepatol (2019) 71(4):731–41. doi: 10.1016/j.jhep.2019.05.015 31173813

[65] Chambers CA Kuhns MS Egen JG Allison JP. Ctla-4-Mediated Inhibition in Regulation of T Cell Responses: Mechanisms and Manipulation in Tumor Immunotherapy. Annu Rev Immunol (2001) 19:565–94. doi: 10.1146/annurev.immunol.19.1.565 11244047

[66] Anderson AC. Tim-3: An Emerging Target in the Cancer Immunotherapy Landscape. Cancer Immunol Res (2014) 2(5):393–8. doi: 10.1158/2326-6066.CIR-14-0039 24795351

[67] Grosso JF Kelleher CC Harris TJ Maris CH Hipkiss EL De Marzo A. Lag-3 Regulates Cd8+ T Cell Accumulation and Effector Function in Murine Self- and Tumor-Tolerance Systems. J Clin Invest (2007) 117(11):3383–92. doi: 10.1172/JCI31184 PMC2000807 17932562

[68] Hasmim M Messai Y Ziani L Thiery J Bouhris JH Noman MZ. Critical Role of Tumor Microenvironment in Shaping Nk Cell Functions: Implication of Hypoxic Stress. Front Immunol (2015) 6:482. doi: 10.3389/fimmu.2015.00482 26441986 PMC4585210

[69] Shi FD Ljunggren HG La Cava A Van Kaer L. Organ-Specific Features of Natural Killer Cells. Nat Rev Immunol (2011) 11(10):658–71. doi: 10.1038/nri3065 PMC3620656 21941294

[70] Kamimura H Yamagiwa S Tsuchiya A Takamura M Matsuda Y Ohkoshi S. Reduced Nkg2d Ligand Expression in Hepatocellular Carcinoma Correlates With Early Recurrence. J Hepatol (2012) 56(2):381–8. doi: 10.1016/j.jhep.2011.06.017 21756848

[71] Vujanovic L Stahl EC Pardee AD Geller DA Tsung A Watkins SC. Tumor-Derived Alpha-Fetoprotein Directly Drives Human Natural Killer-Cell Activation and Subsequent Cell Death. Cancer Immunol Res (2017) 5(6):493–502. doi: 10.1158/2326-6066.CIR-16-0216 28468916

[72] Langhans B Alwan AW Kramer B Glassner A Lutz P Strassburg CP. Regulatory Cd4+ T Cells Modulate the Interaction Between Nk Cells and Hepatic Stellate Cells by Acting on Either Cell Type. J Hepatol (2015) 62(2):398–404. doi: 10.1016/j.jhep.2014.08.038 25195554

[73] Lee UE Friedman SL. Mechanisms of Hepatic Fibrogenesis. Best Pract Res Clin Gastroenterol (2011) 25(2):195–206. doi: 10.1016/j.bpg.2011.02.005 21497738 PMC3079877

[74] Gabele E Brenner DA Rippe RA. Liver Fibrosis: Signals Leading to the Amplification of the Fibrogenic Hepatic Stellate Cell. Front Biosci (2003), 69–77. doi: 10.2741/887 12456323

[75] Baglieri J Brenner DA Kisseleva T. The Role of Fibrosis and Liver-Associated Fibroblasts in the Pathogenesis of Hepatocellular Carcinoma. Int J Mol Sci (2019) 20(7):1723. doi: 10.3390/ijms20071723 PMC6479943 30959975

[76] Hochst B Schildberg FA Sauerborn P Gabel YA Gevensleben H Goltz D. Activated Human Hepatic Stellate Cells Induce Myeloid Derived Suppressor Cells From Peripheral Blood Monocytes in a Cd44-Dependent Fashion. J Hepatol (2013) 59(3):528–35. doi: 10.1016/j.jhep.2013.04.033 23665041

[77] Zhao W Su W Kuang P Zhang L Liu J Yin Z. The Role of Hepatic Stellate Cells in the Regulation of T-Cell Function and the Promotion of Hepatocellular Carcinoma. Int J Oncol (2012) 41(2):457–64. doi: 10.3892/ijo.2012.1497 PMC3582803 22641338

[78] Estevez J Chen VL Podlaha O Li B Le A Vutien P. Differential Serum Cytokine Profiles in Patients With Chronic Hepatitis B, C, and Hepatocellular Carcinoma. Sci Rep (2017) 7(1):11867. doi: 10.1038/s41598-017-11975-7 28928388 PMC5605527

[79] Dondeti MF El-Maadawy EA Talaat RM. Hepatitis-Related Hepatocellular Carcinoma: Insights Into Cytokine Gene Polymorphisms. World J Gastroenterol (2016) 22(30):6800–16. doi: 10.3748/wjg.v22.i30.6800 PMC4974580 27570418

[80] Yi Y He HW Wang JX Cai XY Li YW Zhou J. The Functional Impairment of Hcc-Infiltrating Gammadelta T Cells, Partially Mediated by Regulatory T Cells in a Tgfbeta- and Il-10-Dependent Manner. J Hepatol (2013) 58(5):977–83. doi: 10.1016/j.jhep.2012.12.015 23262246

[81] Zhong M Zhong C Cui W Wang G Zheng G Li L. Induction of Tolerogenic Dendritic Cells by Activated Tgf-Beta/Akt/Smad2 Signaling in Rig-I-Deficient Stemness-High Human Liver Cancer Cells. BMC Cancer (2019) 19(1):439. doi: 10.1186/s12885-019-5670-9 31088527 PMC6515680

[82] Nagao M Nakajima Y Kanehiro H Hisanaga M Aomatsu Y Ko S. The Impact of Interferon Gamma Receptor Expression on the Mechanism of Escape From Host Immune Surveillance in Hepatocellular Carcinoma. Hepatology (2000) 32(3):491–500. doi: 10.1053/jhep.2000.16470 10960440

[83] Faivre S Rimassa L Finn RS. Molecular Therapies for Hcc: Looking Outside the Box. J Hepatol (2020) 72(2):342–52. doi: 10.1016/j.jhep.2019.09.010 31954496

[84] Shi F Shi M Zeng Z Qi RZ Liu ZW Zhang JY. Pd-1 and Pd-L1 Upregulation Promotes Cd8(+) T-Cell Apoptosis and Postoperative Recurrence in Hepatocellular Carcinoma Patients. Int J Cancer (2011) 128(4):887–96. doi: 10.1002/ijc.25397 20473887

[85] Wu K Kryczek I Chen L Zou W Welling TH. Kupffer Cell Suppression of Cd8+ T Cells in Human Hepatocellular Carcinoma Is Mediated by B7-H1/Programmed Death-1 Interactions. Cancer Res (2009) 69(20):8067–75. doi: 10.1158/0008-5472.CAN-09-0901 PMC4397483 19826049

[86] El-Khoueiry AB Sangro B Yau T Crocenzi TS Kudo M Hsu C. Nivolumab in Patients With Advanced Hepatocellular Carcinoma (Checkmate 040): An Open-Label, Non-Comparative, Phase 1/2 Dose Escalation and Expansion Trial. Lancet (2017) 389(10088):2492–502. doi: 10.1016/s0140-6736(17)31046-2 PMC7539326 28434648

[87] Yau T Park J-W Finn RS Cheng A-L Mathurin P Edeline J. Nivolumab Versus Sorafenib in Advanced Hepatocellular Carcinoma (Checkmate 459): A Randomised, Multicentre, Open-Label, Phase 3 Trial. Lancet Oncol (2022) 23(1):77–90. doi: 10.1016/S1470-2045(21)00604-5 34914889

[88] Zhu AX Finn RS Edeline J Cattan S Ogasawara S Palmer D. Pembrolizumab in Patients With Advanced Hepatocellular Carcinoma Previously Treated With Sorafenib (Keynote-224): A Non-Randomised, Open-Label Phase 2 Trial. Lancet Oncol (2018) 19(7):940–52. doi: 10.1016/S1470-2045(18)30351-6 29875066

[89] Kelley RK Sangro B Harris W Ikeda M Okusaka T Kang Y-K. Safety, Efficacy, and Pharmacodynamics of Tremelimumab Plus Durvalumab for Patients With Unresectable Hepatocellular Carcinoma: Randomized Expansion of a Phase I/Ii Study. J Clin Oncol (2021) 39(27):2991–3001. doi: 10.1200/JCO.20.03555 34292792 PMC8445563

[90] Lee DW Cho EJ Lee JH Yu SJ Kim YJ Yoon JH. Phase Ii Study of Avelumab in Patients With Advanced Hepatocellular Carcinoma Previously Treated With Sorafenib. Clin Cancer Res (2021) 27(3):713–8. doi: 10.1158/1078-0432.CCR-20-3094 33139266

[91] Han Y Chen Z Yang Y Jiang Z Gu Y Liu Y. Human Cd14+ Ctla-4+ Regulatory Dendritic Cells Suppress T-Cell Response by Cytotoxic T-Lymphocyte Antigen-4-Dependent Il-10 and Indoleamine-2,3-Dioxygenase Production in Hepatocellular Carcinoma. Hepatology (2014) 59(2):567–79. doi: 10.1002/hep.26694 23960017

[92] Kudo M. Immune Checkpoint Inhibition in Hepatocellular Carcinoma: Basics and Ongoing Clinical Trials. Oncology (2017) 92 Suppl 1:50–62. doi: 10.1159/000451016 28147363

[93] Sangro B Gomez-Martin C de la Mata M Inarrairaegui M Garralda E Barrera P. A Clinical Trial of Ctla-4 Blockade With Tremelimumab in Patients With Hepatocellular Carcinoma and Chronic Hepatitis C. J Hepatol (2013) 59(1):81–8. doi: 10.1016/j.jhep.2013.02.022 23466307

[94] Duffy AG Ulahannan SV Makorova-Rusher O Rahma O Wedemeyer H Pratt D. Tremelimumab in Combination With Ablation in Patients With Advanced Hepatocellular Carcinoma. J Hepatol (2017) 66(3):545–51. doi: 10.1016/j.jhep.2016.10.029 PMC5316490 27816492

[95] Yau T Kang Y-K Kim T-Y El-Khoueiry AB Santoro A Sangro B. Efficacy and Safety of Nivolumab Plus Ipilimumab in Patients With Advanced Hepatocellular Carcinoma Previously Treated With Sorafenib. JAMA Oncol (2020) 6(11):e204564. doi: 10.1001/jamaoncol.2020.4564 33001135 PMC7530824

[96] Kaseb AO Hasanov E Cao HST Xiao L Vauthey J-N Lee SS. Perioperative Nivolumab Monotherapy Versus Nivolumab Plus Ipilimumab in Resectable Hepatocellular Carcinoma: A Randomised, Open-Label, Phase 2 Trial. Lancet Gastroenterol Hepatol (2022) 7(7):208–18. doi: 10.1016/S2468-1253(21)00427-1 PMC8840977 35065057

[97] Koyama S Akbay EA Li YY Herter-Sprie GS Buczkowski KA Richards WG. Adaptive Resistance to Therapeutic Pd-1 Blockade Is Associated With Upregulation of Alternative Immune Checkpoints. Nat Commun (2016) 7:10501. doi: 10.1038/ncomms10501 26883990 PMC4757784

[98] Chu PY Chan SH. Cure the Incurable? Recent Breakthroughs in Immune Checkpoint Blockade for Hepatocellular Carcinoma. Cancers (Basel) (2021) 13(21):5295. doi: 10.3390/cancers13215295 34771459 PMC8582442

[99] Finn RS Ikeda M Zhu AX Sung MW Baron AD Kudo M. Phase Ib Study of Lenvatinib Plus Pembrolizumab in Patients With Unresectable Hepatocellular Carcinoma. J Clin Oncol (2020) 38(26):2960–70. doi: 10.1200/JCO.20.00808 PMC7479760 32716739

[100] Ho WJ Zhu Q Durham J Popovic A Xavier S Leatherman J. Neoadjuvant Cabozantinib and Nivolumab Converts Locally Advanced Hcc Into Resectable Disease With Enhanced Antitumor Immunity. Nat Cancer (2021) 2(9):891–903. doi: 10.1038/s43018-021-00234-4 34796337 PMC8594857

[101] Kelley RK Oliver JW Hazra S Benzaghou F Yau T Cheng A-L. Cabozantinib in Combination With Atezolizumab Versus Sorafenib in Treatment-Naive Advanced Hepatocellular Carcinoma: Cosmic-312 Phase Iii Study Design. Future Oncol (2020) 16(21):1525–36. doi: 10.2217/fon-2020-0283 32491932

[102] Ren Z Xu J Bai Y Xu A Cang S Du C. Sintilimab Plus a Bevacizumab Biosimilar (Ibi305) Versus Sorafenib in Unresectable Hepatocellular Carcinoma (Orient-32): A Randomised, Open-Label, Phase 2–3 Study. Lancet Oncol (2021) 22(7):977–90. doi: 10.1016/s1470-2045(21)00252-7 34143971

[103] Lee MS Ryoo B-Y Hsu C-H Numata K Stein S Verret W. Atezolizumab With or Without Bevacizumab in Unresectable Hepatocellular Carcinoma (Go30140): An Open-Label, Multicentre, Phase 1b Study. Lancet Oncol (2020) 21(6):808–20. doi: 10.1016/s1470-2045(20)30156-x 32502443

[104] Finn RS Qin S Ikeda M Galle PR Ducreux M Kim TY. Atezolizumab Plus Bevacizumab in Unresectable Hepatocellular Carcinoma. N Engl J Med (2020) 382(20):1894–905. doi: 10.1056/NEJMoa1915745 32402160

[105] Huang A Yang XR Chung WY Dennison AR Zhou J. Targeted Therapy for Hepatocellular Carcinoma. Signal Transduct Target Ther (2020) 5(1):146. doi: 10.1038/s41392-020-00264-x 32782275 PMC7419547

[106] Rizzo A Ricci AD. Pd-L1, Tmb, and Other Potential Predictors of Response to Immunotherapy for Hepatocellular Carcinoma: How Can They Assist Drug Clinical Trials? Expert Opin Investig Drugs (2022) 31(4):415–23. doi: 10.1080/13543784.2021.1972969 34429006

[107] Nishina S Hino K. Cd26/Dpp4 as a Therapeutic Target in Nonalcoholic Steatohepatitis Associated Hepatocellular Carcinoma. Cancers (Basel) (2022) 14(2):454. doi: 10.3390/cancers14020454 35053615 PMC8774170

[108] McGranahan N Swanton C. Clonal Heterogeneity and Tumor Evolution: Past, Present, and the Future. Cell (2017) 168(4):613–28. doi: 10.1016/j.cell.2017.01.018 28187284

[109] Kurebayashi Y Ojima H Tsujikawa H Kubota N Maehara J Abe Y. Landscape of Immune Microenvironment in Hepatocellular Carcinoma and Its Additional Impact on Histological and Molecular Classification. Hepatology (2018) 68(3):1025–41. doi: 10.1002/hep.29904 29603348

[110] Zhang Q Lou Y Yang J Wang J Feng J Zhao Y. Integrated Multiomic Analysis Reveals Comprehensive Tumour Heterogeneity and Novel Immunophenotypic Classification in Hepatocellular Carcinomas. Gut (2019) 68(11):2019–31. doi: 10.1136/gutjnl-2019-318912 PMC6839802 31227589

[111] Lin Z Lu D Wei X Wang J Xu X. Heterogeneous Responses in Hepatocellular Carcinoma: The Achilles Heel of Immune Checkpoint Inhibitors. Am J Cancer Res (2020) 10(4):1085–102. PMC7191099 32368387

[112] Atwa SM Odenthal M El Tayebi HM. Genetic Heterogeneity, Therapeutic Hurdle Confronting Sorafenib and Immune Checkpoint Inhibitors in Hepatocellular Carcinoma. Cancers (Basel) (2021) 13(17):4343. doi: 10.3390/cancers13174343 34503153 PMC8430643

[113] Harding JJ Nandakumar S Armenia J Khalil DN Albano M Ly M. Prospective Genotyping of Hepatocellular Carcinoma: Clinical Implications of Next-Generation Sequencing for Matching Patients to Targeted and Immune Therapies. Clin Cancer Res (2019) 25(7):2116–26. doi: 10.1158/1078-0432.CCR-18-2293 PMC6689131 30373752

[114] Kamphorst AO Wieland A Nasti T Yang S Zhang R Barber DL. Rescue of Exhausted Cd8 T Cells by Pd-1-Targeted Therapies Is Cd28-Dependent. Science (2017) 355(6332):1423–7. doi: 10.1126/science.aaf0683 PMC5595217 28280249

[115] Kudo M Finn RS Qin S Han KH Ikeda K Piscaglia F. Lenvatinib Versus Sorafenib in First-Line Treatment of Patients With Unresectable Hepatocellular Carcinoma: A Randomised Phase 3 Non-Inferiority Trial. Lancet (2018) 391(10126):1163–73. doi: 10.1016/S0140-6736(18)30207-1 29433850

[116] Dolladille C Ederhy S Sassier M Cautela J Thuny F Cohen AA. Immune Checkpoint Inhibitor Rechallenge After Immune-Related Adverse Events in Patients With Cancer. JAMA Oncol (2020) 6(6):865–71. doi: 10.1001/jamaoncol.2020.0726 PMC7163782 32297899

[117] Sangro B Chan SL Meyer T Reig M El-Khoueiry A Galle PR. Diagnosis and Management of Toxicities of Immune Checkpoint Inhibitors in Hepatocellular Carcinoma. J Hepatol (2020) 72(2):320–41. doi: 10.1016/j.jhep.2019.10.021 PMC7779342 31954495

[118] Liu Y Wang H Deng J Sun C He Y Zhou C. Toxicity of Tumor Immune Checkpoint Inhibitors-More Attention Should Be Paid. Transl Lung Cancer Res (2019) 8(6):1125–33. doi: 10.21037/tlcr.2019.11.26 PMC6976385 32010590

